# Texture Analysis and Synthesis of Malignant and Benign Mediastinal Lymph Nodes in Patients with Lung Cancer on Computed Tomography

**DOI:** 10.1038/srep43209

**Published:** 2017-02-24

**Authors:** Tuan D. Pham, Yuzuru Watanabe, Mitsunori Higuchi, Hiroyuki Suzuki

**Affiliations:** 1 Linkoping University, Department of Biomedical Engineering, Linkoping, 58183, Sweden; 2 Fukushima Medical University, Department of Regenerative Surgery, Fukushima City, 960-1295, Japan; 3 Fukushima Medical University, Department of Chest Surgery, Fukushima City, 960-1295, Japan

## Abstract

Texture analysis of computed tomography (CT) imaging has been found useful to distinguish subtle differences, which are in- visible to human eyes, between malignant and benign tissues in cancer patients. This study implemented two complementary methods of texture analysis, known as the gray-level co-occurrence matrix (GLCM) and the experimental semivariogram (SV) with an aim to improve the predictive value of evaluating mediastinal lymph nodes in lung cancer. The GLCM was explored with the use of a rich set of its derived features, whereas the SV feature was extracted on real and synthesized CT samples of benign and malignant lymph nodes. A distinct advantage of the computer methodology presented herein is the alleviation of the need for an automated precise segmentation of the lymph nodes. Using the logistic regression model, a sensitivity of 75%, specificity of 90%, and area under curve of 0.89 were obtained in the test population. A tenfold cross-validation of 70% accuracy of classifying between benign and malignant lymph nodes was obtained using the support vector machines as a pattern classifier. These results are higher than those recently reported in literature with similar studies.

Cancer spreads to lymph nodes by occurring in the nodes themselves, which is called lymphoma, or pervades from somewhere else, which is called metastatic cancer. Mediastinal lymph nodes in the proximity of the primary tumor often indicate the first site of metastasis. Most cancer mortality rates are a result of metastasis, and despite its clinical importance, little is known about the genetic and biochemical determinants of metastasis^1^,^2^. The precise detection of lymph node metastasis is therefore a significant contribution to prognoses for many types of cancers^3^.

In medical imaging, PET-CT is a technique using a device that combines both PET scanner and an X-ray CT scanner to sequentially acquire images from both scanners into a co-registered image. Using this technology, functional imaging obtained from PET, which captures the spatial distribution of metabolic or biochemical activity in the body can be aligned with anatomic imaging obtained from CT. For patients suspected with lung cancer, integrated 18F-fluorodeoxyglucose positron emission tomography and/or computed tomography (18F-FDG PET/CT) is a gold standard imaging method performed in hilar and mediastinal lymph node (HMLN) staging of non-small cell lung cancer (NSCLC). Nevertheless, the diagnostic efficiency of PET/CT remains controversial, while another study reveals that the use of raw data of PET/CT is insufficient for assessing mediastinal lymph nodes in patients^4^. A study^5^ found that the false negative rate (1-specificity) of lymph-node metastasis of NSCLC was 13.2%, whereas 45.5% patients were pathologically confirmed as false positive (1-sensitivity), and concluded that lymph node staging using PET-CT is far from being equal to pathological staging. Other findings^6^ on the specificity and sensitivity of PET/CT in detecting HMLN metastases in NSCLC patients were 91.0% and 47.4%, respectively. A recent survey^7^ showed a wide range of the sensitivity of PET-CT in mediastinal LN staging, varying from 40–86.3%, reported by different research groups^8^,^9^,^10^,^11^,^12^,^13^,^14^,^15^, which suggested the limitation of PET-CT for the direct assessment of lymph nodes; and therefore, novel methods are needed to be developed to enhance the reliable evaluation of LN staging of NSCLC^7^. Another study^7^ reported the sensitivity and specificity of PET-CT based on different maximum standardized uptake value (SUVmax) cut-offs with the application of two criteria: 1) the lymph nodes were considered as malignant when their SUVmax was higher than the normal background, and 2) the lymph nodes were considered malignant when their SUVmax was above 2.5. Using criterion 1, the sensitivity and specificity were 48.1% and 88.1%, respectively, in the squamous cell carcinoma (SCC) group, and 57.5% and 95.9%, respectively, in the adenocarcinoma (AC) group. According to criterion 2, the sensitivity and specificity were 37.0% and 90.5%, respectively, in the SCC group, while being 40.0% and 96.3%, respectively, in the AC group. These results showed a significant difference in the sensitivity in both SCC and AC groups, using the two criteria. In particular, the high variability in the false-positive findings was due to the increase in glycolytic activity of benign and inflammatory tissues^16^,^17^,^18^.

Radiomics^19^,^20^,^21^,^22^ refers to the extraction of large amounts of quantitative imaging features from medical images obtained with CT, PET or magnetic resonance imaging (MRI). These features are suggested to be extracted from standard-of-care images, leading to very large useful sources of medical information about cancer patients. The core hypothesis of radiomics is that hidden medical imaging information can be revealed with extracted quantitative features that in turn provide valuable diagnostic, prognostic or predictive assessment^19^. In fact, during the past decades, medical imaging innovations with new instruments and new imaging agents allow the field advancing toward quantitative imaging. Therefore, a need for developing automated and reproducible analysis methodologies to extract more information from image-based features is justified and hold great promises to facilitate better clinical decision making, particularly in the care of patients with cancer at low cost^20^,^21^,^22^. These findings motivated our seeking for advanced image and pattern analysis methods for extracting effective CT features that can better differentiate malignant and benign mediastinal lymph nodes in patients with lung cancer to improve pre-surgical evaluation and clinical decision making.

The highlights of the contribution of this paper are as follows: 1) extraction of texture characteristics of mediastinal lymph nodes on CT using geostatistics can be enhanced with texture synthesis; 2) extraction of texture characteristics of mediastinal lymph nodes on CT using geostatistics can be enhanced with noise addition; and 3) combination of synthesized experimental semivariogram (SV), noise-added SV, and GLCM functions are found to be complementary for differentiating between malignant and benign mediastinal lymph nodes, which results in highest performance in comparison with those obtained from similar studies reported in literature.

## Methods

### Patients and CT

This retrospective study was approved by the institutional review board of Fukushima Medical University, and informed consent was waived. Medical record review was performed in accordance with institutional ethics review board guidelines. Inclusion criteria comprised biopsy-proven primary lung malignancy with pathological mediastinal nodal staging and unenhanced CT of the thorax performed within an interval of less than three months. Studies were performed between April 2010 and April 2015. A total of 148 consecutive patients were included (93 men, 55 women, 36–84 years of age, mean age = 69.41, and median age = 71). There were 105 adenocarcinomas, 28 squamous cell carcinomas, 6 adenosquamous cell carcinomas, 5 large cell carcinomas, 1 small cell lung cancer, 3 pleomorphic carcinomas. Histological analysis was considered gold standard for the diagnosis of benign or malignant nodes, which were obtained in resection specimens (148 patients). Most biopsied lymph nodes were at stations 7 and 4R (no. 4 right lymph nodes). Patients with nodal biopsy more than three months from CT were excluded.

CT studies were obtained on 64 multidetector CT systems (Toshiba Aquilion) with a breath-held helical acquisition of the entire thorax, 135 kV, 180 mAs, 0.50 s/0.5 mm/0.5 × 64 with automatic tube current modulation, table feed, beam pitch 0.640625:1, CTDI (computed tomography dose index) = 94.40–113, and DLP (dose length product) = 3615–3764 mGy. Pathology reports were reviewed to determine the location of nodal biopsy for the selection of corresponding nodes on CT. CT images were reviewed using picture archiving and communication system (PACS) on mediastinal windows settings (width × length = 600 × 100), 5 mm slice thickness reconstruction with no slice overlap, the field of horizontal view with 512 mm diameter and 512 × 512 pixels, and nodes were selected by a thoracic surgeon with 8 years of experience blinded to the pathological result. A total of 271 mediastinal nodes were available for quantitative analysis, all measured less than 20 mm in short axis.

**Figure 1:**
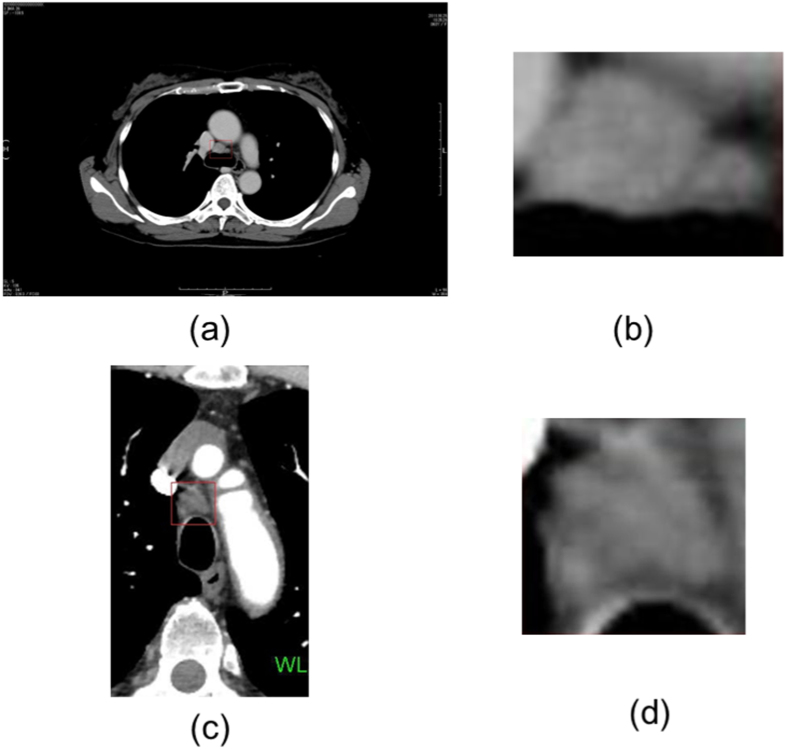
Thoracic lymph nodes shown in rectangular boxes on CT: (**a**) a malignant lymph node and (**b**) its enlarged image, (**c**) a benign lymph node and (**d**) its enlarged image.

As an example, Fig. 1 shows the CT images and the regions of interest (ROIs), which were marked by the thoracic surgeon, of malignant and benign lymph nodes. The enlarged ROIs were automatically extracted to be directly used for texture analysis and texture synthesis without the need of automated image segmentation of the lymph nodes.

### CT Texture Analysis

In order to extract textural information of benign and malignant lymph nodes on two-dimensional (2D) CT (projection from 3D), which were used to train a classifier for the task of pattern recognition, the gray-level co-occurrence matrix (GLCM)^23^ and the semivariogram^24^ features were adopted in this study. The use of these two types of features is based on a rationale that their textural contents are complementary to each other, and therefore textural information redundancy can be avoided. The derivation of the GLCM of an image is based on the statistical dependency on image intensity in the context of image structure, whereas the SV of an image is formulated on the geometrical dependency in the context of statistical difference in image intensity between pixel pairs. The GLCM is mathematically expressed as follows. Let *p* and *q* be be two image intensity levels in an image *I*, an element of the GLCM is defined as


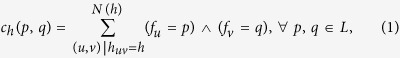


where *f*_*u*_ and *f*_*v*_ are pixels at locations *u* and *v* and having values *p* and *q*, respectively, which are separated by the distance *h*, ∧ stands for the logical AND operator, *L* ∈ *I* is the set of the image intensity levels, and *N(h*) is the total number of pairs of pixels offset by *h*.

Based on Equation (1), the probability of a GLCM element, denoted as *p*_*h*_(*p, q*), can be computed as


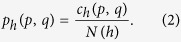


The probabilities of the GLCM defined in Equation (2) allows a variety of definitions of GLCM features. In this study, the following 20 GLCM features were utilized: entropy^23^, energy^23^, correlation^23^, contrast^23^, sum of squares (variance)^23^, sum average^23^, sum variance^23^, sum entropy^23^, difference variance^23^, difference entropy^23^, information measures of correlation^23^, autocorrelation^25^, dissimilarity^25^, homogeneity^25^, cluster prominence^25^, cluster shade^25^, maximum probability^25^, inverse difference^26^, inverse difference normalized^26^, and inverse difference moment normalized^26^.

The mathematical expression of the SV is described as follows. Let *f(u*) be a regionalized variable of an image *I* at location *u*, which represents an image intensity value at *u*, the SV, denoted as *γ(h*), which expresses the characteristics of the regionalized variables is defined as^24^


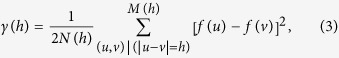


where *f(v*) is the image intensity at *v*, locations *u, v* ∈ *I* are separated by a lag or distance *h*, and *M(h*) is the total number of pairs of the regionalized variables separated by *h*.

It has recently been found that adding noise at some certain level to the SV can help enhance the textural characteristics^27^. Therefore, additive white Gaussian noise was also added to the CT ROIs containing the lymph nodes to improve the discriminative power of the SV texture.

### CT Texture Synthesis

The reason for resorting to the application of texture synthesis in this study is to solve the problem of small-size images of mediastinal lymph nodes on CT. If the small-size images can be enlarged while the statistical characteristics of their appearance can be reserved, then texture features of the lymph nodes can be better extracted to be subsequently used as training data for machine-learning algorithms. In fact, texture has been well recognized as one of most important features of medical imaging data. We therefore employ texture synthesis to statistically enlarge the regions of interest containing the lymph nodes in order to enhance texture analysis.

Texture synthesis can be stated as follows: given a smaller texture image, texture synthesis can generate a larger synthesized one that appears to be generated by the same underlying stochastic process of the smaller one^28^. In other word, the purpose of texture synthesis is to construct a larger image being as similar as possible to textural characteristics of the given sample. The texture synthesis algorithm developed in ref. 29 was adopted in this study, because it is one of the most popular synthesis algorithms^30^. This algorithm works as follows. Let *w(f*) be an *n* × *n* window (patch), where *f* is the pixel at the center, *I* the original image, *S* the synthesized image, and *d*[*w(f*_*I*_), *w(f*_*S*_)] a distance between the neighboring pixels of *f*_*I*_ (*f* ∈ *I*) and *f*_*S*_ (*f* ∈ *S*) within the corresponding windows. The minimum distance from *w(f*_*S*_) to all *w(f*_*I*_) is


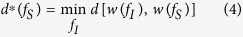


The set of pixels similar to *f*_*S*_, denoted as 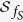, are defined by


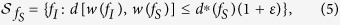


where *ε* is a tolerance value. Synthesized pixels are then sequentially generated by randomly selecting a pixel from 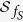 each time.

**Figure 2:**
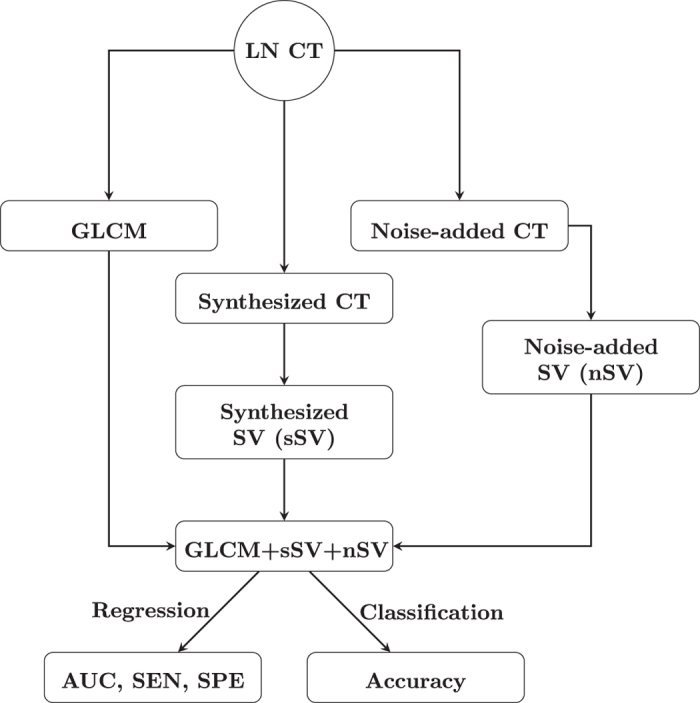
Flow chart of CT texture analysis and synthesis of malignant and benign lymph-node CT (LN CT) to compute area under curve (AUC), sensitivity (SEN), specificity (SPE), and classification accuracy using combination of features of gray-level co-occurrence (GLCM), synthesized semivariogram (sSV), and noise-added semivariogram (nSV).

Figure 2 graphically shows the pipeline procedure of texture analysis and synthesis to obtain the area under curve of the receiver operating characteristic, sensitivity, specificity, and accuracy with respect to the classification of benign and malignant mediastinal lymph nodes on CT.

## Results

A set of 133 malignant mediastinal lymph nodes, and a set of 138 benign mediastinal lymph nodes of the patients were used in this experiment to test the effectiveness of the combination of the GLCM and SV features to differentiate between the two sets of samples. Instead of carrying out the segmentation of the lymph nodes, all CT regions of interest that were manually marked by an experienced thoracic surgeon were automatically extracted for feature extraction. The image sizes of the malignant regions are between about 16 × 24 pixels to 84 × 85 pixels. The image sizes of the benign regions are between about 26 × 18 pixels to 122 × 112 pixels.

Table 1 shows the results of the texture analysis and synthesis in terms of the receiver operating characteristics (ROC), obtained from the logistic regression model using the binomial distribution and the logit function, which is the inverse of the logistic function (S-shaped curve). Two sets of GLCM features were used in the experiment: 4 features (contrast, correlation, energy, homogeneity), and all 20 features described earlier. The GLCM features were computed using a publically available Matlab program^31^. To reduce the computational time in the calculation of the GLCM in several directions, in this study, the direction for extracting GLCM features was based on the row-wise distance of one pixel, which is the pixel next to the pixel of interest on the same row. For the extraction of texture using the semivariogram (SV), the first 10 lags (*h* = 1, …, 10) and the first 30 lags (*h* = 1, …, 30) were used. The lags of the SV were computed in the horizontal and vertical directions of the image.

With the implementation of 4 GLCM features, the AUC = 0.76, which is smaller than the AUC = 0.81 with the use of 20 GLCM features. The AUCs of the semivariogram without noise (SV) and with additive white Gaussian noise of zero mean and 0.02 variance (nSV) with 10 lags are the same (0.72), but both have reversely different sensitivity (SEN) and specificity (SPE): SEN = 55% and SPE = 81% obtained for SV, and SEN = 72% and SPE = 66% for nSV. The calculation of the SV of the CT samples were limited because of the small sample sizes. Therefore the image samples were synthesized to enlarge their sizes, and the SV of the synthesized images were obtained with larger lags: 20 lags with a synthesized size of 200 × 200 pixels and 30 lags with a synthesized size of 250 × 250 pixels, denoted as sSV1 and sSV2, respectively. The texture synthesis parameters for the patch size and overlapping size (overlapping bar between patches) were set to 10 × 10 and 6, respectively, and the tolerance *ε* = 0.1 as suggested in ref. 29. The distance *d* was used as the convolution of the normalized sum of squared differences metric and a two-dimensional Gaussian kernel^29^. Being shown in Table 1, the GLCM (4 and 20 features) and SV (SV, nSV, sSV1, sSV2) features were combined in several different ways to compute the AUC, SEN, and SPE using the logistic regression model.

Let malignant and benign lymph nodes be positive and negative cases, respectively. Instances of true positive, true negative, false negative, and false positive are denoted as TP, TN, FN, and FP, respectively. The optimal operating threshold for the ROC curve was determined by finding the slope *m* using the following equation^32^:


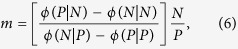


where *ϕ(N*|*P*) is the cost of misclassifying a malignant lymph node (LN) as a benign LN, *ϕ(P*|*N*) is the cost of misclassifying a benign LN as a malignant LN, *P* = *TP* + *FN*, and *N* = *TN* + *FP*. In this study, *ϕ(P*|*P*) = 0, *ϕ(N*|*P*) = 0.5, *ϕ(P*|*N*) = 0.5, and *ϕ(N*|*N*) = 0. The optimal operating point was found by moving the straight line with slope *m* from the upper left corner of the ROC plot, where false-positive rate (x-axis) = 0 and true-positive rate (y-axis) = 1, down and to the right, until it intersects the ROC curve.

**Figure 3:**
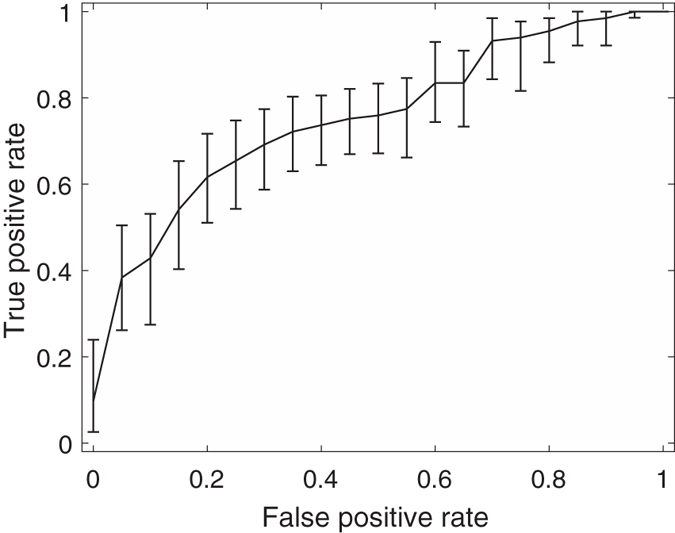
Receiver operating curve (ROC) with pointwise 95% confidence bounds obtained from 4 GLCM features of malignant and benign mediastinal lymph nodes in patients with lung cancer on computed tomography, where the area under curve (AUC) = 0.76 with *p*-value < 0.0001.

**Figure 4:**
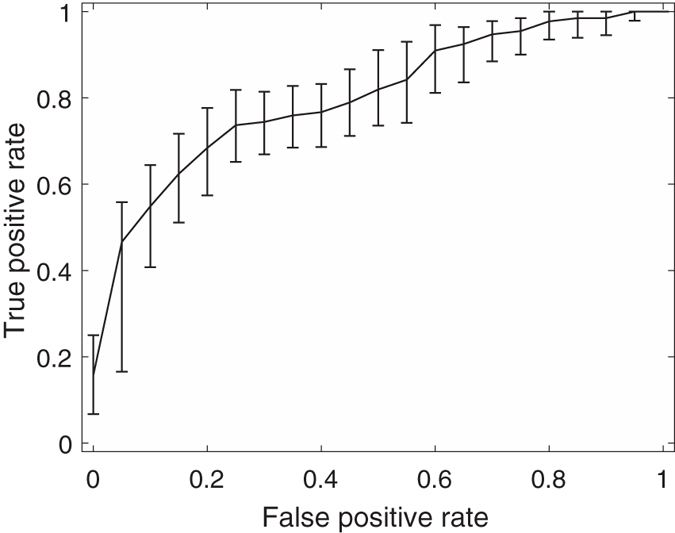
Receiver operating curve (ROC)with pointwise 95% confidence bounds obtained from 20 GLCM features of malignant and benign mediastinal lymph nodes in patients with lung cancer on computed tomography, where the area under curve (AUC) = 0.81 with *p*-value < 0.0001.

**Figure 5:**
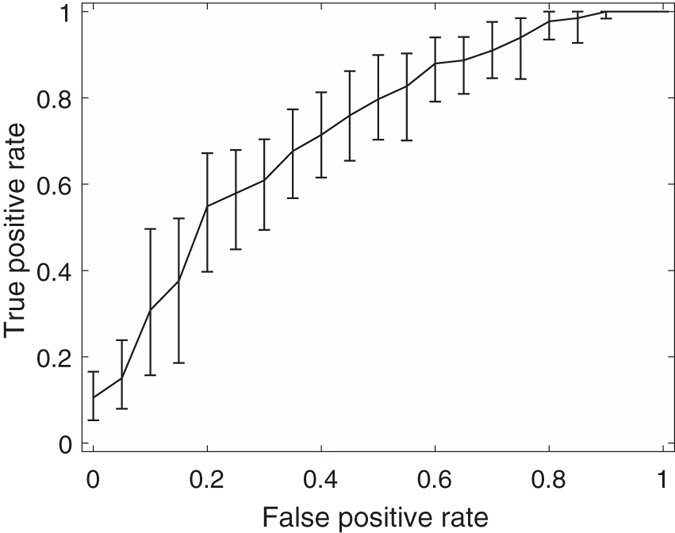
Receiver operating curve (ROC) with pointwise 95% confidence bounds obtained from the first 10 lags of the semivariogram of malignant and benign mediastinal lymph nodes in patients with lung cancer on computed tomography, where the area under curve (AUC) = 0.72 with *p*-value < 0.0001.

**Figure 6:**
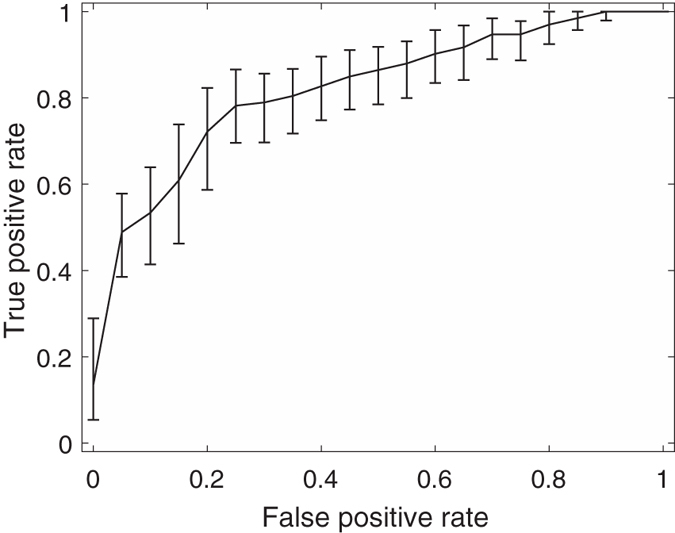
Receiver operating curve (ROC) with pointwise 95% confidence bounds obtained from the combination of 20 GLCM features and first 10 lags of the semivariogram of malignant and benign mediastinal lymph nodes in patients with lung cancer on computed tomography, where the area under curve (AUC) = 0.82 with *p*-value < 0.0001.

**Figure 7:**
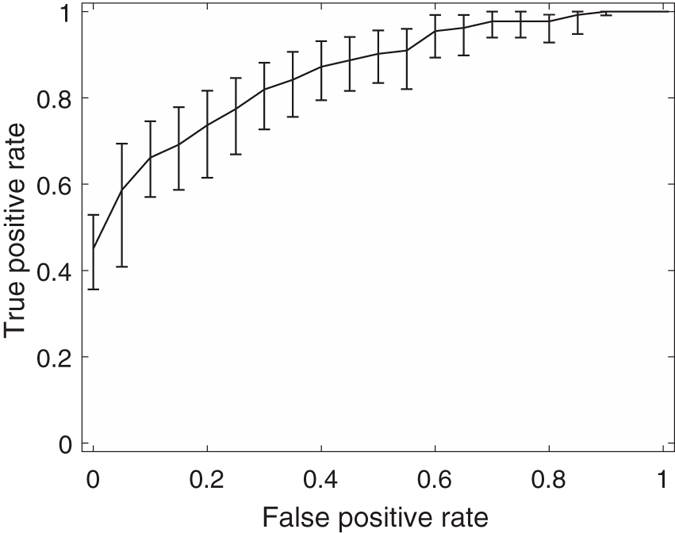
Receiver operating curve (ROC) with pointwise 95% confidence bounds obtained from combined GLCM (4 features) and noise-added semivariogram (30 lags) analysis and synthesis (250 × 250) of malignant and benign mediastinal lymph nodes in patients with lung cancer on computed tomography, where the area under curve (AUC) = 0.86 with *p*-value < 0.0001.

**Figure 8:**
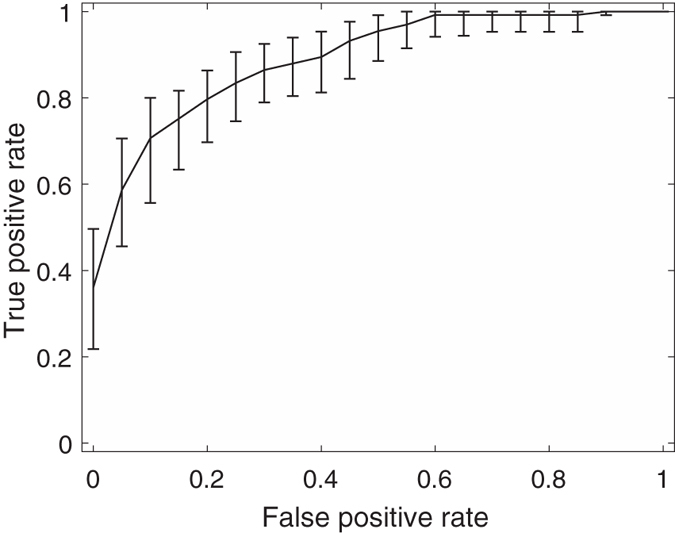
Receiver operating curve (ROC) with pointwise 95% confidence bounds obtained from combined GLCM (20 features) and noise-added semivariogram (30 lags) analysis and synthesis (250 × 250) of malignant and benign mediastinal lymph nodes in patients with lung cancer on computed tomography, where the area under curve (AUC) = 0.89 with *p*-value < 0.0001.

Among these feature combinations, the combine feature set of 20 GLCM features, nSV and sSV2 yields the best results with AUC = 0.89, SEN = 75%, and SPE = 90%. Figures 3, 4, 5, 6, 7 and 8 show the plots of the ROC curves obtained from 4 GLCM features, 20 GLCM features, SV with the first 10 lags, the combination of 20 GLCM features and SV with the first 10 lags, the combination of 4 GLCM features and additive white Gaussian noise (zero mean and 0.02 variance)-added synthesized SV, and the combination of 20 GLCM features and additive white Gaussian noise (zero mean and 0.02 variance)-added synthesized SV, respectively. Figures 3, 4, 5, 6, 7 and 8 also show the pointwise 95% confidence intervals using vertical averaging that takes the vertical values of the ROC curves for fixed false-positive rates and averages the corresponding true-positive rates^33^, and sampling using bootstrap with 1000 replicas for computing 95% confidence intervals.

Table 2 shows the tenfold cross-validation results obtained from the support-vector-machines (SVM), naive-Bayes (NB), and linear-discriminant-analysis (LDA) classifiers, using the GLCM, SV, nSV, sSV2, and their combinations. The SVM classifier gives the best result (70%) for the combination of GLCM, nSV and sSV2 features (70%), which has the highest AUC (0.89). The LDA yields the best results for the use of both 4 and 20 GLCM features (72% and 70%, respectively), the combination of 20 GLCM and SV features (70%), the combination of 20 GLCM and nSV features (71%), and the combination of 4 GLCM, nSV, and sSV2 features (66%). The NB classifier provides the best accuracy for the use of the SV texture (65%).

## Discussion

In a similar and recent study^34^, it was reported that the combination of GLCM, run-length matrix, and shape features achieved the best AUC of 0.87, 81% sensitivity, and 80% specificity using the logistic regression model, and 71% SVM-based accuracy for the classification of benign and malignant mediastinal lymph nodes in lung-cancer patients. Another similar and most recent study^35^ on CT texture analysis of benign and malignant mediastinal lymph nodes in patients with non-small-cell lung carcinoma applied wavelet analysis to extract fine and coarse textures within the regions of interest (ROIs), and by using the logistic regression, the AUC of 0.8, 53% sensitivity, and 97% specificity were obtained.

The results reported in this paper were obtained without the need of precise identification of the lymph nodes, which therefore relieves the burden of implementing an image segmentation algorithm as a pre-processing step for feature extraction. In principle, GLCM is computed in terms of the numbers of pixel pairs for specific intensity levels, while the SV is obtained in terms of the average of the intensity difference between pixel pairs for specific distances. These two statistical approaches are therefore complimentary for extracting textural information on CT of malignant and benign mediastinal lymph nodes in patients with lung cancer. The AUC of 0.89 with 75% sensitivity, 90% specificity, and 70% SVM-based classification accuracy suggest the potential power of the combination of the two approaches for the discrimination of malignant and benign lymph nodes on CT data.

The current results confirm the counter-intuitive idea that the addition of noise to CT images at some certain level, which is the white Gaussian noise with zero mean and 0.02 variance in this study, can enhance the discriminative power of the textural characteristics within the ROIs. The results also confirm that texture synthesis can be useful for computing the SV function with larger lags, where inherently small image sizes of the lymph nodes hinder such computation and classification performance. In this report, only CT textural information of the malignant and benign lymph nodes using the complementary combination of the GLCM and SV was explored. The investigation of other texture synthesis algorithms and the inclusion of other sources of image information about the lymph nodes are expected to improve the predictive power of discriminating between benign and malignant lymph nodes in lung cancer.

It is known that 18F-FDG PET/CT is a standard procedure performed in patients with suspected lung cancer for identifying mediastinal lymph node staging of NSCLC^5^,^35^. However, the diagnostic power of the use of PET/CT imaging measured in terms of sensitivity and specificity is well below pathological findings, and this challenging issue is due to the increase in glycolytic activity of benign and inflammatory tissue that makes it difficult to reduce the false-positive diagnosis results^5^. An attempt was made to apply texture analysis and machine learning with SVM on F18-FDG PET/CT images to differentiate between benign and malignant bone and soft-tissue lesions^36^. Therefore, texture analysis and synthesis of lymph nodes in patients with lung cancer on both PET and CT images to extract textural information about the effect of the metabolism of F18-FDG are promising to provide a potential non-invasive procedure for improving the predictive value of F18-FDG PET/CT.

In this study, we used both metastatic and non-metastatic lymph nodes from the same patients for the CT-based texture analysis and synthesis. Because we could not obtain a sufficient number of samples from each patient for the comparison between per-patient and per-node results, and also the definite diagnosis of histological subtypes of the lymph nodes before surgery was not always possible, we analyzed all the lymph nodes obtained together from all the patients. In general, non-metastatic lymph nodes in patients with squamous cell carcinoma are larger than those with other histological subtypes. Furthermore, there was no previous evidence showing differences in imaging parameters among the histological subtypes, including texture. In spite of that, the results reported in this study did not take into account the size of the lymph nodes, thus showing the promising power of the proposed method.

## Conclusion

Texture analysis and synthesis of mediastinal lymph nodes on CT obtained from a relatively large number of patients with lung cancer have been presented and discussed in the foregoing sections. An AUC of 0.89 based on regression analysis, and accuracy of 70% based on the tenfold cross-validation of SVM-classified results were achieved and shown to be superior to those of similar studies reported in current literature.

The radiological features provided by both GLCM and SV appear to be complementary to each other, and worth further investigating to provide improvement by taking into account other spatial orientations for feature extraction while keeping a balance of the computational burden in terms of computer speed and memory. Applications of more effective algorithms for texture synthesis as well as methods for estimating optimal levels of noise addition for enhancing texture characteristics of lymph nodes on CT would certainly increase the classification accuracy. Finally, exploration of recently developed machine-learning and signal processing techniques, such as deep learning^37^ and sparse-dictionary learning^38^, would contribute to improving the differentiation between malignant and benign lymph nodes.

## Additional Information

**How to cite this article:** Pham, T. D. *et al*. Texture Analysis and Synthesis of Malignant and Benign Mediastinal Lymph Nodes in Patients with Lung Cancer on Computed Tomography. *Sci. Rep.* **7**, 43209; doi: 10.1038/srep43209 (2017).

**Publisher's note:** Springer Nature remains neutral with regard to jurisdictional claims in published maps and institutional affiliations.

## Figures and Tables

**Table 1:** Receiver operating characteristics (AUC = area under curve, SEN = sensitivity, SPE = specificity) obtained from generalized linear regression model, using gray-level co-occurrence matrix (GLCM), semivariogram (SV), noise-added semivariogram (nSV), and synthesized semivariogram (sSV) features, where sSV1 has 20 lags of synthesized image size = 200 × 200, and sSV2 has 30 lags of synthesized image size = 250 × 250.

Texture	AUC	*p*-value	SEN (%)	SPE (%)
GLCM (4 features)	0.76	<0.0001	59	83
GLCM (20 features)	0.81	<0.0001	73	78
SV (10 lags)	0.72	<0.0001	55	81
nSV (10 lags)	0.72	<0.0001	72	66
GLCM (20 features) + SV (10 lags)	0.82	<0.0001	78	76
GLCM (20 features) + nSV (10 lags)	0.85	<0.0001	77	80
GLCM (20 features) + nSV (10 lags) + sSV1	0.86	<0.0001	63	95
GLCM (4 features) + nSV (10 lags) + sSV2	0.86	<0.0001	73	86
GLCM (20 features) + nSV (10 lags) + sSV2	0.89	<0.0001	75	90

**Table 2:** Tenfold cross-validation results (%) obtained from support vector machines (SVM), naive Bayes (NB), and linear discriminant analysis (LDA), using gray-level co-occurrence matrix (GLCM), semivariogram (SV), noise-added semivariogram (nSV), and synthesized semivariogram (sSV) features, where synthesized image size = 250 × 250.

Texture	SVM	NB	LDA
GLCM (4 features)	68	66	72
GLCM (20 features)	66	66	70
SV (*h* = 10)	64	65	63
GLCM (20 features) + SV (10 lags)	67	67	70
GLCM (20 features) + nSV (10 lags)	69	66	71
GLCM (4 features) + nSV (10 lags) + sSV2 (30 lags)	65	59	66
GLCM (20 features) + nSV (10 lags) + sSV2 (30 lags)	70	62	68
